# Soluble Urokinase Plasminogen Activator Receptor (suPAR): Role in the Risk Stratification of Potentially Infected Patients Presenting at the Emergency Department

**DOI:** 10.3390/diagnostics16071076

**Published:** 2026-04-02

**Authors:** Matteo Baldetti, Silvia Velocci, Riccardo Belardi, Vito Nicola Di Lecce, Carla Paganelli, Nicola Antonio Fortunato, Teresa Ricciardulli, Alessandro Terrinoni, Massimo Pieri, Sergio Bernardini, Jacopo Maria Legramante, Marilena Minieri

**Affiliations:** 1 Unit of Laboratory Medicine, University Hospital Tor Vergata, 00133 Rome, Italy; matteobaldetti@gmail.com (M.B.); velocci.silvia@gmail.com (S.V.); belardiriccardo92@gmail.com (R.B.); alessandro.terrinoni@uniroma2.it (A.T.); massimo.pieri@ptvonline.it (M.P.); bernards@uniroma2.it (S.B.); marilenami@gmail.com (M.M.); 2 Emergency Department, University Hospital Tor Vergata, 00133 Rome, Italy; vitonicola.dilecce@ptvonline.it (V.N.D.L.); carla.paganelli@ptvonline.it (C.P.); nicolaantonio.fortunato@ptvonline.it (N.A.F.); teresa.ricciardulli@ptvonline.it (T.R.); 3 Department of Systems Medicine, University of Rome Tor Vergata, 00133 Rome, Italy; 4 Department of Experimental Medicine, University of Rome Tor Vergata, 00133 Rome, Italy

**Keywords:** suPAR, emergency department, sepsis, inflammation, prognosis, biomarkers, clinical biochemistry

## Abstract

**Background/Objectives**: Risk stratification of patients presenting to the emergency department (ED) with suspected infection is essential. Soluble urokinase plasminogen activator receptor (suPAR) has emerged as a promising biomarker of immune activation and outcome prediction, but its role in febrile ED patients remains to be fully defined. The aim of this study was to evaluate the prognostic value of suPAR and its potential clinical application in the ED. **Methods**: A single-center retrospective study including 125 patients was performed. Plasma suPAR levels were measured together with C-reactive protein (CRP) and procalcitonin (PCT), and their association with 28-day mortality using Cox regression and ROC curve analyses was assessed. **Results**: SuPAR was independently associated with the risk of 28-day mortality, showing a very high negative predictive value. Higher suPAR levels were associated with an increased risk of death. **Conclusions**: SuPAR may represent a valuable tool to support early patient assessment and risk stratification in the ED, potentially improving resource utilization and patient prioritization.

## 1. Introduction

Sepsis has been declared a worldwide health concern, and it is continuing to be a major cause of morbidity and death globally, resulting in a heavy financial and economic impact. For instance, in high-income countries, such as the United States (US), cost per patient for sepsis care is estimated to be between $20,000 to $50,000 because of expenses related to hospitalization, medication, diagnostic tests, and more [1]. From an epidemiological point of view, data published in 2017 showed that the estimated cases of sepsis worldwide were 48.9 million, with 11 million sepsis-related deaths. After 4 years, in 2021, 166 million sepsis cases were estimated, with 21.4 million of all-cause sepsis-related deaths reported, accounting for 31.5% of global deaths [2,3]. While the concept of sepsis has its roots in ancient times, the latest definitions for sepsis, septic shock, and organ dysfunction were established in 2016. Sepsis is defined as a life-threatening condition caused by a dysregulated host response to infection, while septic shock is characterized by the need for vasopressors to maintain a mean arterial pressure (MAP) above 65 mmHg. Organ dysfunction, considered a consequence of infection, is now assessed using the Sequential Organ Failure Assessment (SOFA) score [4]. Several biomarkers, such as procalcitonin (PCT) and C-reactive protein (CRP), have been studied so far for their potential roles in diagnosis, prognostication, treatment guidance, and early recognition of organ dysfunction in sepsis [5,6,7,8]. Soluble urokinase plasminogen activator receptor (suPAR) is released upon cleavage of its membrane-bound form (uPAR) and participates in several cellular processes, including cell adhesion, chemotaxis, and coagulation [9]. Levels of this biomarker have been shown to correlate with several inflammatory biomarkers, including C-reactive protein (CRP), interleukin-6 (IL-6), and tumor necrosis factor-alpha (TNF-α), supporting its role as a marker of immune activation. CRP appears to show stronger correlations with IL-6, fibrinogen, and erythrocyte sedimentation rate than suPAR does, likely reflecting its role as a classical acute-phase reactant. Conversely, suPAR may be more indicative of systemic immune activation rather than a purely acute-phase response, also exhibiting baseline sex-based differences, with women generally showing higher circulating levels than men [9,10,11,12]. In contrast to inflammatory cytokines such as TNF-α, which show transient fluctuations in circulation, suPAR is also characterized by high stability in plasma and minimal circadian variation, making it a promising biomarker for clinical use [13]. Clinically, suPAR levels are associated with worse prognosis across various conditions, including sepsis and infections of different origins, cancer, and cardiovascular diseases [14,15,16,17,18,19]. This evidence supports the concept that suPAR could be a possible applicable prognostic biomarker in the setting of emergency medicine. Nowadays, it is crucial to identify markers that can reliably help to speed up the rule-out process for patients coming to the ED, thereby freeing resources and making room for new individuals who require immediate care, in an attempt to face the crucial problem of emergency room overcrowding. The aim of this study is to evaluate the 28-day mortality prediction capability of suPAR, alone and in combination with other inflammatory and infection markers, such as Procalcitonin (PCT) and C-Reactive Protein (CRP), in potentially infected patients presenting to the ED with fever. The goal is to determine the potential [7] of suPAR as a valuable tool able to help clinicians to identify low-risk patients, improve patient flow, and optimize the use of ED resources for new patients.

## 2. Materials and Methods

### 2.1. Study Design and Operations

The study, conducted at the ED of Tor Vergata University Hospital in Rome, is an observational, retrospective, single-center study. We evaluated the clinical utility of specific biomarkers for prognostic purposes in adult patients presenting with fever and possible infection in the ED. A specific panel of laboratory tests named “Fever Package”, which included CRP and PCT, was created and triggered for every selected patient. Successively, suPAR analysis was performed in selected patients with suspected infection to retrospectively evaluate early prognosis through mortality risk assessment. While PCT and CRP were measured in serum samples as part of routine clinical testing, suPAR levels were determined in plasma samples obtained from residual specimens collected upon ED admission. A database including ED admission date, 28-day follow-up, outcome at admission, death or re-hospitalization, comorbidities, PCT, CRP, and suPAR levels, as well as the type of infection identified (blood cultures, oropharyngeal and anorectal swabs, urine cultures, cerebrospinal fluid, and chest imaging) was created. All patients were followed up by phone at 28 days to assess clinical outcomes, allowing correlation of baseline biomarker levels with disease progression, treatment response, and mortality, and enabling evaluation of biomarker performance in a real-world ED setting. Fever at presentation was defined as a temperature > 37.8 °C.

### 2.2. Stratification Strategy and Testing

A total of 125 patients were selected as “potentially septic” and levels of CRP, PCT, and suPAR were determined for all of them. Patients were also stratified based on comorbidities such as lung disease, cardiovascular disease, diabetes, hypertension, age, and sex.

### 2.3. Statistical Analysis

The primary endpoint of the study was overall 28-day mortality from ED admission. Continuous variables were expressed as mean (standard deviation) or median (interquartile range), depending on the data distribution, and compared using Student’s *t*-test or the Mann–Whitney U test, as appropriate.

Associations between candidate variables and endpoints were evaluated using Cox regression analyses, both univariate and multivariate, and hazard ratios (HRs) were calculated. Survivors were compared with non-survivors.

The discriminative ability of the analyzed variables for predicting mortality was assessed using receiver operating characteristic (ROC) curve analysis, with determination of the area under the ROC curve (AUC). For regression analyses, variables were dichotomized according to threshold values obtained from this study using the Youden index derived from ROC curve analysis.

For each biomarker, sensitivity, specificity, positive and negative predictive values (PPV, NPV), and positive and negative likelihood ratios (LR+, LR−) were reported, along with 95% confidence intervals (CI) for mortality.

All analyses were performed using R Studio software (Posit team (2025). RStudio: Integrated Development Environment for R. Posit Software2024.12.1+563, PBC, Boston, MA, USA, URL: http://www.posit.co/). Two-sided *p*-values < 0.05 were considered statistically significant. Variables that were statistically significant in the univariate analysis were included in the multivariate analysis.

## 3. Results

Demographic and clinical characteristics are summarized in Table 1. A total of 125 patients were selected. Among these, 66 (52.8%) were males and 59 (47.2%) were females. Mean age was 65.9 ± 17.9 years old, with hypertension (44%), cardiovascular disease (36.8%), and renal disease (22.4%) being the most frequent comorbidities in the study population. The nature of the infections was also assessed. There was a higher prevalence of bacterial infections compared to viral infections. Moreover, in the bacterial infection group, 24 died within 30 days. Patients suffering from viral infections were 20, and they all survived.

Based on the comparison between the different groups (deceased vs. survivors) and the *p*-values obtained, no statistically significant differences were observed for most variables. However, three comorbidities—renal disease, cardiovascular disease, and hypertension—were significantly more prevalent in deceased patients compared to survivors. Additionally, age was significantly higher among deceased patients than among survivors.

Interestingly, the mean suPAR levels were 8.85 ng/mL for viral infections and 9.57 ng/mL for bacterial infections. Welch’s *t*-test showed a statistically significant difference between the two groups, although the absolute difference was modest.

At triage, CRP and PCT in the “Fever Package” were both measured, and after selection of patients potentially infected, suPAR levels were measured. Descriptive statistics of the test results in our patient population are summarized in Table 2.

Our findings show that there is a significant difference between the median levels of suPAR and PCT between deceased and survivors.

Based on the current literature, differences exist between males and females in circulating suPAR levels. For this reason, a sex-based comparison in our population was performed, and results were summarized in Table 3.

Our results show no significant sex-based differences in the circulating levels of suPAR.

To further explore potential differences in the prognostic performance of the respective sex-based cut-offs, an ROC analysis was performed for both males and females separately. The AUC was 0.74 for males and 0.76 for females. Using Youden’s index, the optimal cut-offs were 11.85 ng/mL for males and 11.15 ng/mL for females. Although a slight numerical difference between sex-specific thresholds was observed, the magnitude of this difference was small.

We then continued to assess the stability of both sex-based and unique cut-offs through a bootstrapping technique to determine which one to adopt for further analyses. Results are summarized in Table 4.

Our findings show that the male cut-off shows the greatest variability (SD = 1.99, 95% CI = 7.25–14.3), suggesting limited stability. The female cut-off appears more stable (SD = 1.39, 95% CI = 6.45–14.4), while the overall cut-off is the most reliable (SD = 0.99, 95% CI = 10.8–14.25), indicating that aggregating the data reduces variability.

The previous findings prompted us to perform a Cox univariate analysis to assess the prediction capabilities of the comorbidities and biomarkers that were considered, using the most stable cut-off from a statistical standpoint. Table 5 summarizes the hazard ratios (HRs), along with the cut-offs.

Statistically significant results were obtained for age, cardiovascular disease, renal disease, suPAR, CRP, and PCT. Interestingly, the HR for age is 1.06, meaning that the risk of death increases by 6% with each additional year of age. Patients with cardiovascular disease had a 3.8-fold higher risk of death compared to those without. Notably, patients with suPAR levels above the cut-off (11.15 ng/mL) showed an 8.29-fold increase in the risk of death, highlighting the strong prognostic value of this marker in the ED setting. Significant associations were also found for PCT and CRP levels above their respective cut-offs, although the risk increase was not as pronounced as for suPAR.

The significant findings from the univariate analysis prompted us to perform a multivariate Cox analysis to adjust for potential confounders, and the results are summarized in Table 6.

The adjustment caused the other biomarkers and the considered co-morbidities to lose statistical significance, such as cardiovascular disease and renal disease. Moreover, the results now showed that any unitary increase in the levels of suPAR is associated with an increased death risk of 1.19-fold (19%). Age showed a similar result, with an increased death risk of 1.07-fold (7%). Overall, these results show age and, most importantly, suPAR can act as an independent predictor of 28-day mortality, even in the presence of co-morbidities.

Additionally, sensitivity and specificity for each one of the biomarkers considered and in their combined form were assessed, and results were summarized in Table 6.

**Figure 1:**
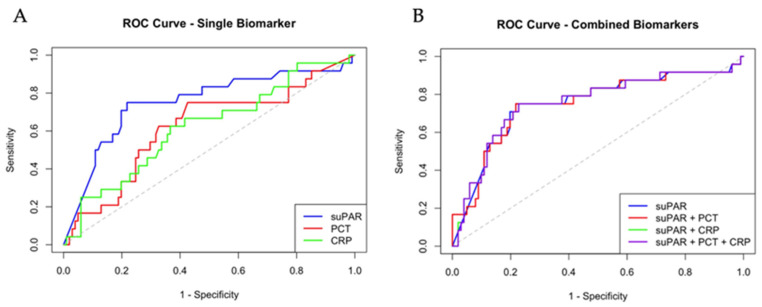
**ROC curve illustration of the single and combined biomarkers.** (**A**) Area under the curve (AUC) associated with each biomarker (suPAR, PCT, and CRP) considered individually. While PCT and CRP showed comparable AUC values, suPAR demonstrated the highest AUC. (**B**) Combining suPAR with both CRP and PCT did not substantially change the AUC ROC curves were compared using DeLong’s test, and *p*-values for comparisons between AUCs were calculated (suPAR vs. PCT, *p*-value 0.03; suPAR vs. CRP, *p*-value 0.04).

SuPAR exhibited the highest specificity compared to CRP and PCT, highlighting its superior ability to correctly identify surviving patients. The sensitivity was 75%, similar to PCT, but the AUC was higher (0.75), as shown in Figure 1A. In contrast, CRP had the lowest sensitivity among the markers considered, and PCT showed the lowest specificity. Additionally, PCT had a 28-day mortality negative predictive value (NPV) of 91%, very close to suPAR at 93%. The combined model integrating all three biomarkers did not substantially change either the negative or positive predictive values. On one hand, these data indicate that suPAR had the highest NPV (93%) among all biomarkers considered, proving itself to be a superior and independent biomarker to rule out 28-day mortality. On the other hand, suPAR’s ability to predict the event of death over 28 days is lower, as our results showed a 45% PPV.

Subsequently, we performed an ROC curve on combinations of the three biomarkers analyzed to evaluate possible improvements in the 28-day mortality prediction ability. Figure 1A and Table 7 show that AUC and NPV are not particularly influenced when PCT and CRP are evaluated in a combined model with suPAR.

## 4. Discussion

The use of biomarkers, at the time of ED admission for risk assessment in patients with suspected infections (particularly pulmonary infections and other morbidities), has been extensively documented [5,6,7,8,20,21,22,23]. Current literature reports that soluble urokinase Plasminogen Activator Receptor (suPAR) is an emergent biomarker with a very promising prognostic value in different clinical conditions and, more specifically, in an ED setting for potentially infected patients [24,25,26,27,28,29,30,31]. Even though the value of suPAR has also been studied in septic patients, to date, there are no studies whose population was based on patients selected because of a febrile state (>37.8 °C), with no other inclusion criteria. The aim of the present study was to evaluate the ability of a sepsis-related and emergent biomarker, suPAR, to predict 28-day mortality in patients accessing the ED triage with signs of fever and selected as “potentially septic”. In our cohort, circulating suPAR levels did not differ significantly between males and females. Although the calculated sex-specific cut-offs were slightly different (males: 11.85 ng/mL; females: 11.15 ng/mL), the absolute difference was minimal and unlikely to be clinically relevant. Through bootstrapping, the male-specific cut-off showed greater variability (SD 1.99, 95% CI 7.25–14.3) compared to the female-specific (SD 1.39, 95% CI 6.45–14.4) and the overall cut-off (SD 0.99, 95% CI 10.8–14.25). These findings support the use of a universal threshold of 11.15 ng/mL within the context of this study, which appears more stable and reliable for identifying patients with elevated suPAR levels, thereby minimizing misclassification due to potential sampling variability rather than true sex-based biological differences and allowing for conservative risk stratification. Overall, our data do not support the implementation of sex-specific cut-offs for suPAR in this clinical setting; however, further studies with larger sample sizes are warranted to better evaluate this issue. The present study also demonstrated that suPAR (u 0.75; *p*-value < 0.05) was better than CRP and PCT in discerning between deceased and surviving people after 28 days, confirming the results reported in the current literature [30,32]. Another study showed suPAR being able to predict mortality in 28 days through a 12 ng/mL cut-off with an NPV of 88.3% in patients with one point on the quick Sequential Organ Failure Assessment (qSOFA) [25]. SuPAR was even proven to be more reliable than CRP and PCT in estimating the risk of mortality (NPV 93%; *p*-value < 0.05) in patients with values below the 11.15 ng/mL cut-off. The study by Schultz et al., in which suPAR NPV was calculated to be 100% over 7 days in a non-selected ED patient population, generated similar findings [29]. These results have significant implications for the clinician decision-making process, as well as for the management of time and hospital resources in providing patient care. Notably, the combination of the three biomarkers considered did not significantly modify the hazard ratio (HR), indicating that CRP and PCT provide minimal additional prognostic value compared with suPAR when estimating mortality risk per unit increase. In multivariate analysis, adjusting for potential confounders such as renal and cardiovascular diseases, suPAR remained a valuable independent predictor of mortality. Importantly, our results also highlight the relative limitations of CRP and PCT in this context, as they were less effective in identifying patients at high risk of short-term mortality. Additionally, only a limited number of studies have specifically investigated the pathogen-discriminating ability of suPAR. A meta-analysis pooling data from nine studies, including a total of 1237 patients, reported that suPAR achieved an AUC of 0.82 for the diagnosis of bacterial infections [33]. However, this analysis did not further evaluate its capacity to differentiate among specific types of pathogens. Moreover, another study demonstrated that suPAR is not capable of reliably distinguishing between viral and bacterial infections [33,34]. Conversely, our results showed a statistically significant difference in circulating suPAR mean levels between patients with viral and bacterial infections. Specifically, the bacterial group had higher average suPAR levels than the viral group, although the absolute difference was modest. In our cohort, all patients with viral infections (*n* = 20) survived, and suPAR levels in this group were generally lower than in bacterial infections. SuPAR appears to reflect overall disease severity rather than being pathogen-specific, and differences between groups likely mirror differences in clinical severity rather than the type of infection. Although this study was conducted in a single-center hospital setting, it provides valuable insights for primary care practice. Indeed, patients presenting to the ED with either measured or reported fever often represent inappropriate approaches, that is, patients who would benefit more from basic diagnostic and therapeutic resources available in primary care rather than hospital facilities. The high negative predictive value of suPAR could serve as a potential useful tool for general practitioners when deciding whether to manage febrile patients by themselves or refer them to the hospital ED. Rapid assays for suPAR measurement, including lateral-flow point-of-care tests capable of providing results within minutes, are increasingly available and may facilitate its implementation in both emergency and primary care settings [35,36]. This choice could rely on the information provided by suPAR biomarkers to make safer and more responsible decisions regarding patient care, thereby potentially reducing inappropriate ED admissions. Furthermore, the immediate assessment after presentation to the ED through suPAR level determination could indeed help the clinician in optimizing the use of hospital resources, patient prioritization, and improving patient flow through the ED. The present study has several limitations. First, its retrospective design may have introduced selection bias. Second, the relatively small sample size, derived from a single-center cohort, and the limited number of mortality events (*n* = 24) may have reduced the statistical power of the multivariate Cox analysis and increased the risk of model overfitting. Therefore, the observed association between suPAR levels and mortality should be interpreted with caution. Larger multicenter studies including a greater number of patients are needed to confirm these findings and enhance their generalizability. In addition, a broader cohort with a more detailed characterization of infection type would provide a stronger basis to evaluate whether the prognostic value of suPAR differs according to the site of infection (e.g., pulmonary, abdominal, cutaneous, urinary) and to further explore its potential role in distinguishing between viral and bacterial infections.

## 5. Conclusions

The use of suPAR is increasingly recognized as a valuable tool during the initial assessment of patients with suspected infection, as it aids in risk stratification and provides clinicians with information useful for managing time, effort, and resources in the ED. Our study confirmed that this biomarker is an excellent independent predictor of 28-day mortality and is highly effective in identifying low-risk patients presenting to the ED with a febrile state. The use of this biomarker also carries important clinical and economic implications, as it may facilitate a faster “rule-out” of low-risk patients, allowing more serious patients to be admitted more efficiently. This could lead to significant cost savings by optimizing resource allocation and streamlining patient flow in the ED. Further studies are required to stabilize the knowledge associated with the routine use of suPAR in an ED setting.

## Figures and Tables

**Table 1:** **Characteristics of the study population.** Values expressed as percentages (%) indicate the proportion of patients within each group for each variable. Data are presented as mean (standard deviation, SD) where specified. The Chi-square test (χ^2^) was used to assess significance between groups for categorical variables, while Student’s *t*-test was used for comparisons of age. The symbol (*) is used to indicate statistically significant results (*p*-value ≤ 0.05).

	Total (*N* = 125)	Deceased (*N* = 24)	Survivors (*N* = 101)	*p*-Value
**Age**				
Age, mean (SD)	65.9 (17.9)	77.1 (11.6)	63 (18.1)	0.001 *
**Sex**				
Males, *N* (%)	66 (52.8)	14 (58.3)	52 (51.4)	0.55
Females, *N* (%)	59 (47.2)	10 (41.6)	49 (48.5)	0.55
**Comorbidities**				
Hypertension *N* (%)	55 (44)	15 (62.5)	40 (39.6)	0.04 *
Diabetes *N* (%)	26 (20.8)	7 (29.1)	19 (18.8)	0.26
Lung Disease, *N* (%)	15 (12)	2 (8.3)	13 (12.8)	0.54
Cardiovascular Disease, *N* (%)	46 (36.8)	16 (66.6)	30 (29.7)	0.01 *
Cancer, *N* (%)	19 (15.2)	4 (16.6)	15 (14.8)	0.82
Renal Disease, *N* (%)	28 (22.4)	11 (45.8)	17 (16.8)	0.002 *
**Clinical Syndrome at ED access**				
Respiratory, *N* (%)	73 (58.4)	14 (58.3)	59 (58.4)	0.99
Urinary Tract Infection	16 (12.8)	2 (8.3)	14 (13.8)	0.47
Abdominal	11 (8.8)	2 (8.3)	9 (8.9)	0.93
Skin	2 (1.6)	0	2 (1.9)	0.49
Sepsis	21 (16.8)	6 (25)	15 (14.8)	0.23
Central Nervous System	2 (1.6)	1 (4.1)	1 (0.9)	0.27
**Nature of the infections**				
Viral	20	0	20	
Bacterial	104	24	80	
Protozoan	1	0	1	

**Table 2:** **Summary of CRP, PCT, and suPAR plasma levels.** Data are presented as median and the difference between first quartile (Q1) and third quartile (Q3) of the biomarker’s concentration. The symbol (*) is used to indicate statistically significant results (*p*-value ≤ 0.05).

	Total	Deceased	Survivors	*p*-Value
**suPAR (ng/mL)**				
Median	8	14.25	7	<0.001 *
(Q1–Q3)	5.6–12.3	9.05–16	5.36–10.36	
**CRP (mg/L)**				
Median	53.8	86.05	52	0.142
(Q1–Q3)	30.15–127.5	35.48–218.60	27.2–124.5	
**PCT (ng/mL)**				
Median	0.15	0.59	0.12	0.014 *
(Q1–Q3)	0.05–0.72	0.17–1.95	0.05–0.51	

**Table 3:** **Sex-based comparison of circulating suPAR levels.** Data is presented as the mean value of circulating suPAR levels in males and females. Mann–Whitney–Wilcoxon was performed as distribution of suPAR levels in the study population was not normal.

	Total	Males	Females
**suPAR (ng/mL)**			
Mean	9.02	9.03	9.01
Median (IQR)	8 (6.70)	8 (6.60)	8 (6.95)

**Table 4:** **Bootstrapping results on suPAR cut-offs.** Bootstrapping analysis was performed by randomly resampling and recalculating the cut-offs using Youden’s index. Median, mean, SD (Standard Deviation), and CI (Confidence intervals) at 95% confidence were calculated as stability indicators of the respective cut-offs.

Group	Median	Mean	SD	95% CI
Males	11.85	11.03	1.99	7.25–14.3
Females	11.15	11.46	1.39	6.45–14.40
Total	11.15	11.45	0.99	10.8–14.25

**Table 5:** **Univariate Cox regression analysis of biomarkers and clinical characteristics for the prediction of 28-day mortality.** Biomarker cut-off values were determined from Receiver Operating Characteristic (ROC) curves using Youden’s index. The symbol (*) is used to indicate statistically significant results (*p*-value ≤ 0.05).

Variable	Cut-Off	HR (95% CI)	*p*-Value
Age		1.06 (1.10–1.02)	0.0013 *
Sex		1.31 (2.94–0.58)	0.5183
Hypertension		2.18 (4.98–0.95)	0.0647
Diabetes		1.65 (3.99–0.69)	0.2627
Lung Disease		0.65 (2.78–0.15)	0.5649
Cardiovascular Disease		3.80 (8.88–1.62)	0.0021 *
Cancer		1.20 (3.52–0.41)	0.7362
Renal Disease		3.47 (7.74–1.55)	0.0024 *
suPAR (ng/mL)	11.15	8.29 (20.93–3.28)	0.000008 *
CRP (mg/L)	77.4	2.49 (5.68–1.09)	0.0308 *
PCT (ng/mL)	0.159	3.47 (8.74–1.38)	0.0083 *

CI, confidence intervals.

**Table 6:** **Cox multivariate analysis on combined biomarkers and comorbidities models.** The table shows the HR obtained through a multivariate model, highlighting the significance of the findings through the *p*-value. The concordance of the model is 0.83, and the Wald test indicates that the global model is statistically significant. The symbol (*) is used to indicate statistically significant results (*p*-value ≤ 0.05).

Variable	HR (95% CI)	*p*-Value
suPAR	1.18 (1.047–1.331)	0.0067 *
PCR	1.001 (0.996–1.006)	0.6652
PCT	1.028 (0.961–1.1)	0.421
Cardiovascular Disease	1.875 (0.751–4.68)	0.1778
Renal Disease	1.783 (0.768–4.144)	0.1786
Age	1.058 (1.017–1.101)	0.0055 *

CI, confidence intervals.

**Table 7:** **ROC and AUC analyses results of the single and combined biomarkers.** The table reports the diagnostic performance of each biomarker. Positive and negative predictive values, as well as likelihood ratios, were calculated from the confusion matrix derived from true and false positives and negatives. The optimal cut-off for each marker was determined using the Youden index.

Model	Threshold	AUC (95% CI)	Sensitivity	Specificity	PPV (95% CI)	NPV (95% CI)	LR+ (95% CI)	LR− (95% CI)
suPAR (95% CI)	11.15	0.751 (0.628–0.874)	75%	78%	45% (0.293–0.615)	93% (0.853–0.974)	3.443 (2.227–5.324)	0.32 (0.159–0.644)
CRP (95% CI)	77.4	0.62 (0.491–0.748)	62%	63%	28% (0.171–0.431)	87% (0.779–0.942)	1.706 (1.141–2.551)	0.592 (0.346–1.013)
PCT (95% CI)	0.159	0.62 (0.488–0.752)	75%	57%	30% (0.185–0.426)	90% (0.807–0.965)	1.762 (1.275–2.435)	0.435 (0.213–0.888)
suPAR + CRP (95% CI)	0.223	0.755 (0.631–0.878)	75%	77%	44% (0.285–0.603)	93% (0.851–0.973)	3.293 (2.149–5.048)	0.324 (0.161–0.653)
suPAR + PCT (95% CI)	0.224	0.75 (0.626–0.874)	75%	78%	45% (0.293–0.615)	93% (0.853–0.974)	3.443 (2.227–5.324)	0.32 (0.159–0.644)
suPAR + PCT + CRP (95% CI)	0.223	0.754 (0.631–0.878)	75%	77%	44% (0.285–0.603)	93% (0.851–0.973)	3.293 (2.149–5.048)	0.324 (0.161–0.653)

CI, confidence intervals; PPV, positive predictive value; NPV, negative predictive value; LR, likelihood ratio; AUC, area under curve.

## Data Availability

Data collected and analyzed for the purposes of this study are not made available for sharing as they contain sensitive patient information.

## Abbreviations

The following abbreviations are used in this manuscript:

HR	Hazard Ratio
ED	Emergency Department
PCT	Procalcitonin
CRP	C-Reactive Protein
suPAR	Soluble Urokinase Plasminogen Activator Receptor
